# A comprehensive study on classification of COVID-19 on computed tomography with pretrained convolutional neural networks

**DOI:** 10.1038/s41598-020-74164-z

**Published:** 2020-10-09

**Authors:** Tuan D. Pham

**Affiliations:** Center for Artificial Intelligence, Prince Mohammad Bin Fahd University, Khobar, 31952 Saudi Arabia

**Keywords:** Viral infection, Computer science

## Abstract

The use of imaging data has been reported to be useful for rapid diagnosis of COVID-19. Although computed tomography (CT) scans show a variety of signs caused by the viral infection, given a large amount of images, these visual features are difficult and can take a long time to be recognized by radiologists. Artificial intelligence methods for automated classification of COVID-19 on CT scans have been found to be very promising. However, current investigation of pretrained convolutional neural networks (CNNs) for COVID-19 diagnosis using CT data is limited. This study presents an investigation on 16 pretrained CNNs for classification of COVID-19 using a large public database of CT scans collected from COVID-19 patients and non-COVID-19 subjects. The results show that, using only 6 epochs for training, the CNNs achieved very high performance on the classification task. Among the 16 CNNs, DenseNet-201, which is the deepest net, is the best in terms of accuracy, balance between sensitivity and specificity, $F_1$ score, and area under curve. Furthermore, the implementation of transfer learning with the direct input of whole image slices and without the use of data augmentation provided better classification rates than the use of data augmentation. Such a finding alleviates the task of data augmentation and manual extraction of regions of interest on CT images, which are adopted by current implementation of deep-learning models for COVID-19 classification.

## Introduction

Image findings have been increasingly recognized as a useful tool for rapid diagnosis of COVID-19^1^. The use of chest computed tomography (CT) to detect COVID-19 symptoms has been reported to have high sensitivity and can predate positive tests carried out in a laboratory^2–7^. Latest articles on image analysis of COVID-19 can be further found at the European Radiology webaite^8^. Because of the potential utilization of CT data, hospitals overloaded with admissions of patients are using CT imaging to diagnose COVID-19 infection and to decide the order of treatment of infected patients. In fact, chest CT has an important role in urgent clinical assessment and decision making for treatment of COVID-19 patients who suffer from severe and worsening respiratory symptoms. In other words, CT scans can be used to assess the severity of the infected lungs as well as progress of the disease, which tremendously help medical doctors in curbing the virus. It has been suggest that examinations and reports of CT findings can be used as a basis for improving the quality of care for COVID-19 patents^9^.

While CT imaging is useful for the diagnosis of COVID-19, manual reading of the scans is time-consuming and subject to human error. Therefore, the need for advanced artificial intelligence (AI)-based automated image analysis has the potential to analyze CT scans in the assessment of COVID-19. AI-based image analysis methods can provide accurate and rapid diagnosis of the disease to cope with the demand for a large number of patients^10^. For example, a manual assessment of a CT scan can take up to 15 minutes, while AI-based image analysis requires only a few seconds. Furthermore, AI can be useful in improving clinical workflow efficiency^11^.

A recent baseline study on AI for automated classification of COVID-19 using the largest publicly available CT dataset was reported in^12^, which will be described subsequently as the database used in this study. This work used the pretrained DenseNet for the classification task. These authors adopted transfer learning and data augmentation for training the pretrained deep-learning model with new image data. The rationale is that transfer learning can relieve the need for acquiring a large amount of training data by reusing a developed model as the starting point for training a new model with a different task. The data augmentation was performed by using a large dataset of chest X-ray images. The rationale for image data augmentation is to increase the size of the training dataset with plausible examples in order to improve the performance and ability of the deep-learning model to generalize the power of classification by getting familiar with samples of high variance.

Another recent work reported on the use of ten pretrained CNNs for classifying CT scans of COVID-19 and non-COVID-19 subjects^13^. These authors reported that ResNet-101 and Xception provided the best classification results on training and testing a CT dataset consisting 106 COVID-19 patients and 86 non-COVID-19 subjects. The CNNs were trained and tested with regions of interest extracted from the CT scans that were defined by a radiologist.

Other previous works on the classification of COVID-19 on CT scans were reported in^14–16^. A 3D deep-learning network was developed for the detection of COVID-19 from 4356 3D chest CT scans obtained from 3322 patients^14^. The network extracted both 2D local and 3D global features from the CT scans. This network, called COVNet, was built on the pretrained RestNet50. In^15^, the pretrained Inception was modified to detect COVID-19 using extracted regions of interest on CT scans obtained from 180 cases of COVID-19 and 79 cases of SARs-COV-2. In^16^, a total of 618 CT scans were used, consisting of 219 CT scans from 110 COVID-19 patients, 224 CT scans from 224 patients with Influenza-A viral pneumonia, and 175 CT scans from healthy people. Pulmonary regions of interest were extracted from the CT scans, and pretrained ResNet-18 was used for image feature extraction. Finally, the Noisy-or Bayesian function was used to classify the image regions into three types: COVID-19, Influenza-A-viral-pneumonia, and irrelevant-to-infection.

However, it should be noted that the CT datasets used in the studies reported in^13–16^ are not publicly available. In this study, a comprehensive investigation on 16 pretrained CNNs for classification of COVID-19 using a publicly available CT database is presented. These pretrained CNNs reflect a variety of computational complexity and accuracy based on the training and testing of the ImageNet database^17^. Findings of this investigation would facilitate the timely deployment of AI-assisted tools to hospitals and clinics in terms of ease of both data preparation and software implementation for fighting against the pandemic.

## Methods

### COVID-19 CT database

**Figure 1:**
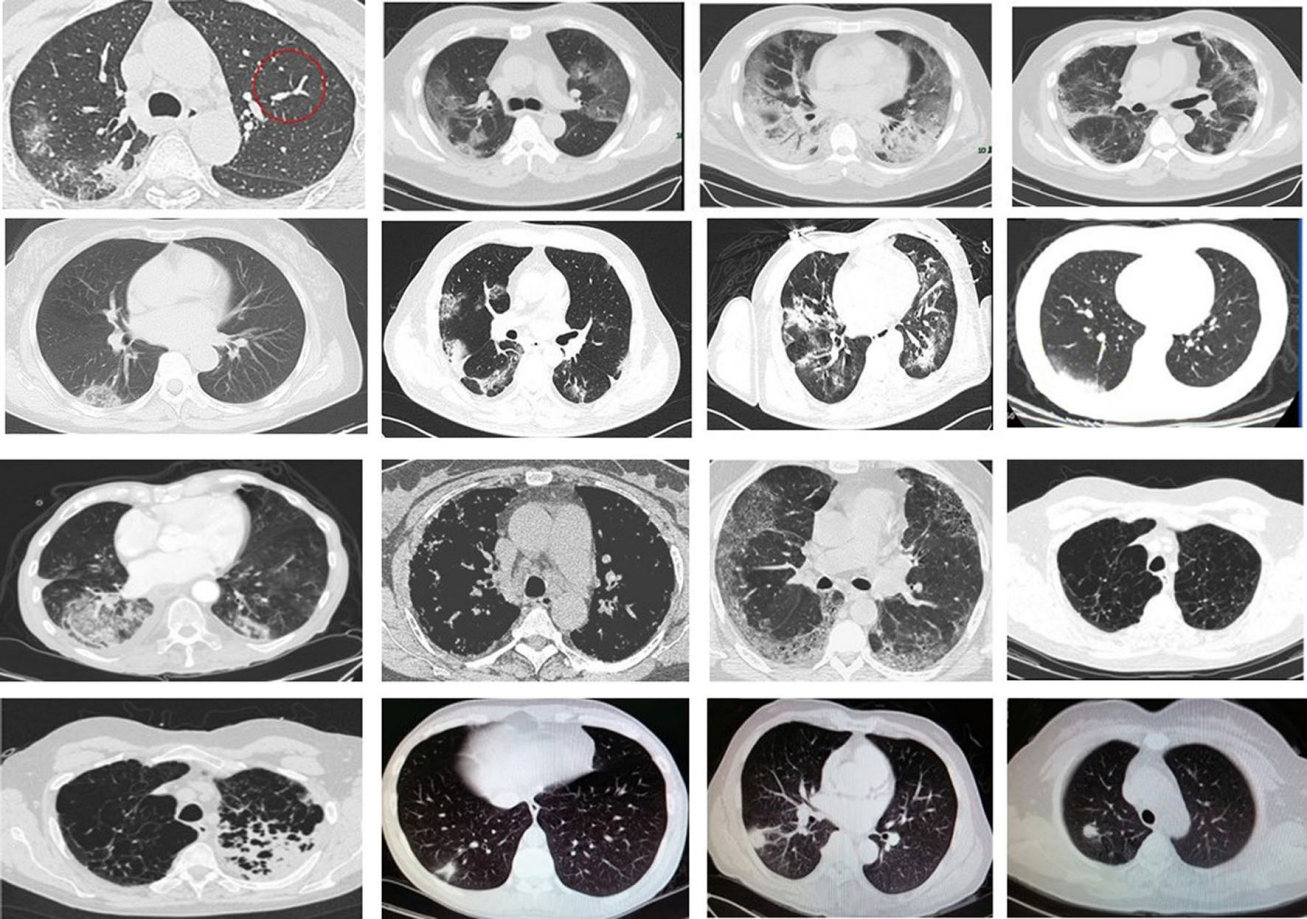
CT images: Rows 1 and 2: COVID-19, Rows 3 and 4: non-COVID-19 (aspect ratios of some images were rescaled to fit the figure frame).

The COVID-19 CT database used in this study is publicly available^18^, and its details are described in^12^. The database consists of 349 CT images containing clinical findings of COVID-19 from 216 patients, and 397 CT images obtained from non-COVID-19 subjects. These CT images were collected from COVID19-related papers published in medRxiv, bioRxiv, NEJM, JAMA, Lancet, and others. Figure 1 shows CT images of COVID-19 and non-COVID-19. The usefulness of this dataset has been confirmed by a senior radiologist of Tongji Hospital, Wuhan, China. The radiologist has performed diagnosis and treatment of a large number of COVID-19 patients during the virus outbreak between January and April 2020^18^.

### Implementation of pretrained CNNs

Sixteen pretrained CNNs were investigated in this study for the classification of whole CT images to differentiate COVID-19 from non-COVID-19. These networks are: (1) SqueezeNet, (2) GoogLeNet, (3) Inception-v3, (4) DenseNet-201, (5) MobileNet-v2, (6) ResNet-18, (7) ResNet-50, (8) ResNet-101, (9) Xception, (10) Inception-ResNet-v2, (11) ShuffleNet, (12) NasNet-Mobile, (13) NasNet-Large, (14) AlexNet, (15) VGG-16, and (16) VGG-19. These pretrained networks were trained on more than a million images from the ImageNet database^17^. The pretrained networks can classify images into 1000 object categories, such as keyboard, mouse, pencil, and many animals. As a result, these networks have learned rich features representing a wide range of images. The properties of these networks are described in Table 1. To enable the reproduction of the results reported in this study, configurations for the transfer learning are described as follows.

**Table 1:** Properties of 16 pre-trained CNNs.

CNN	Depth	Size (MB)	Parameters (millions)	Input image size
AlexNet	8	227	61.0	227 $\times$ 227
GoogLeNet	22	27	7.0	224 $\times$ 224
SqueezeNet	18	4.6	1.24	227 $\times$ 227
ShuffleNet	50	6.3	1.4	224 $\times$ 224
ResNet-18	18	44	11.7	224 $\times$ 224
ResNet-50	50	96	25.6	224 $\times$ 224
ResNet-101	101	167	44.6	224 $\times$ 224
Xception	71	85	22.9	299 $\times$ 299
Inception-v3	48	89	23.9	299 $\times$ 299
Inception-ResNet-v2	164	209	55.9	299 $\times$ 299
VGG-16	16	515	138	224 $\times$ 224
VGG-19	19	535	144	224 $\times$ 224
DenseNet-201	201	77	20.0	224 $\times$ 224
MobileNet-v2	53	13	3.5	224 $\times$ 224
NasNet-Mobile	*	20	5.3	224 $\times$ 224
NasNet-Large	*	360	88.9	331 $\times$ 331

*indicates NASNet-Mobile and NasNetLarge networks do not consist of a linear sequence of modules.

First, the layer graph from the pretrained network was extracted. If the network was a SeriesNetwork object, such as AlexNet, VGG-16, or VGG-19, then the list of layers was converted to a layer graph. In most pretrained networks, the last layer with learnable weights is a fully connected layer. This fully connected layer was replaced with a new fully connected layer with the number of outputs equal to the number of classes in the new data set, which is 2, in this study. In some pretrained networks, such as SqueezeNet, the last learnable layer is a 1-by-1 convolutional layer instead. In this case, the convolutional layer was replaced with a new convolutional layer with the number of filters equal to the number of classes.

For the option of data augmentation in this study, random reflection, translation, and scaling were carried out. Random reflection was done in the top-bottom direction, where each image was reflected vertically with probability = 0.5. The range of horizontal translation applied to the input image = [− 30, 30], where the translation distance was measured in pixels. The horizontal translation distance was selected randomly from a continuous uniform distribution within the specified range. Similarly, the interval of vertical translation applied to the input image in pixels = [− 30, 30]. The vertical translation distance was selected randomly from a continuous uniform distribution within the specified interval. The range of horizontal scaling was applied to the input image, where the horizontal scale factor was selected randomly from a continuous uniform distribution within the specified interval = [0.9, 1.1]. Similarly, the range of vertical scaling was applied to the input image, where the vertical scale factor was selected randomly from a continuous uniform distribution within the specified interval = [0.9, 1.1].

The original whole CT images were converted into RGB images and resized to fit into the input image size of each pretrained CNN. For the training options, the stochastic gradient descent with momentum optimizer was used, where the momentum value = 0.9000; gradient threshold method = $L_2$ norm; minimum batch size = 10; maximum number of epochs = 6; initial learning rate = 0.0003; the learning rate remained constant throughout training; the training data were shuffled before each training epoch, and the validation data were shuffled before each network validation; and factor for $L_2$ regularization (weight decay) = 0.0001.

### Statistical measures of classification performance

Five statistical measures used for evaluating the two-class classification performance of the pretrained CNNs are accuracy, sensitivity, specificity, $F_1$ score, and the area under the receiver operating characteristic (ROC) curve (AUC).

Let the sensitivity (*SEN*) be the percentage of COVID-19 patients who are correctly identified as having the infection, and expressed as

1 $$\begin{aligned} SEN = \frac{TP}{P} \times 100 = \frac{TP}{TP+FN} \times 100, \end{aligned}$$

where *TP* is called true positive, denoting the number of COVID-19 patients who are correctly identified as having the infection, *FN* false negative, denoting the number of COVID-19 patients who are misclassified as having no infection of COVID-19, and *P* the total number of COVID-19 patients.

Let the specificity (*SPE*) be the percentage of non-COVID-19 subjects who are correctly classified as having no infection of COVID-19:

2 $$\begin{aligned} SPE = \frac{TN}{N} \times 100 = \frac{TN}{TN+FP} \times 100, \end{aligned}$$

where *TN* is called true negative and denotes the number of non-COVID-19 subjects who are correctly identified as having no infection of COVID-19, *FP* false positive, denoting the number of non-COVID-19 subjects who are misclassified as having the infection, and *N* the total number of non-COVID-19 subjects.

The percent accuracy (*ACC*) of the classification is defined as

3 $$\begin{aligned} ACC = \frac{TP+TN}{P+N} \times 100. \end{aligned}$$

The $F_1$ score is defined as the balance between precision (TP divided by TP and FP) and sensitivity:

4 $$\begin{aligned} F_1 = \frac{2TP}{2TP+FP+FN}. \end{aligned}$$

The ROC is a probability curve created by plotting the TP rate against the FP rate at various threshold settings, and the AUC represents the measure of performance of a classifier. The AUC value is within the range between 0.5 and 1, where the value = 0.5 represents the performance of a random classifier and the value = 1 indicates a perfect one. Thus, the higher the AUC is, the better the classifier performs. The AUC was calculated using the trapezoidal integration to estimate the area under the ROC curve.

## Results

To compare the results with those obtained from previous reports, the dataset was randomly split into 80% for training and 20% for testing. The data splitting was repeated 5 times to obtain the average and standard deviation for each CNN. The whole CT images were used as the data input, which were resized to fit the input image size of each pretrained CNN, in both training and testing phases. The network training was performed for with and without data augmentation. Tables 2 and 3 show the classification results obtained from the 16 CNNs with and without data augmentation, respectively.

**Table 2:** Classification results with data augmentation.

CNN model	Accuracy (%)	Sensitivity (%)	Specificity (%)	$F_1$ score	AUC
AlexNet	74.50 ± 4.40	70.46 ± 6.37	79.05 ± 8.61	0.75 ± 0.04	0.83 ± 0.04
GoogLeNet	78.97 ± 3.70	75.95 ± 13.69	82.38 ± 10.53	0.79 ± 0.06	0.91 ± 0.04
SqueezeNet	78.52 ± 7.56	91.56 ± 7.63	63.81 ± 23.79	0.82 ± 0.04	0.90 ± 0.01
ShuffleNet	86.13 ± 10.16	83.54 ± 19.89	89.05 ± 5.77	0.86 ± 0.12	0.93 ± 0.06
ResNet-18	90.16 ± 2.36	89.45 ± 7.31	90.95 ± 9.29	0.91 ± 0.02	0.96 ± 0.05
ResNet-50	92.62 ± 4.19	91.14 ± 3.35	94.29 ± 5.15	0.93 ± 0.04	0.98 ± 0.01
ResNet-101	89.71 ± 10.05	82.28 ± 20.09	98.10 ± 2.18	0.89 ± 0.12	0.97 ± 0.03
Xception	85.68 ± 6.76	90.72 ± 4.79	80.00 ± 19.64	0.87 ± 0.05	0.94 ± 0.04
Inception-v3	91.28 ± 8.25	90.30 ± 5.12	92.38 ± 11.98	0.92 ± 0.08	0.97 ± 0.02
Inception-ResNet-v2	86.35 ± 5.71	88.19 ± 6.37	84.29 ± 14.50	0.87 ± 0.05	0.95 ± 0.05
VGG-16	78.52 ± 10.02	74.68 ± 30.14	82.86 ± 15.91	0.76 ± 0.17	0.91 ± 0.04
VGG-19	83.22 ± 5.85	90.72 ± 3.19	74.76 ± 12.96	0.85 ± 0.04	0.90 ± 0.05
DenseNet-201	91.72 ± 6.52	88.61 ± 8.86	95.24 ± 4.36	0.92 ± 0.07	0.97 ± 0.03
MobileNet-v2	87.25 ± 10.46	95.78 ± 2.64	77.62 ± 21.63	0.89 ± 0.08	0.95 ± 0.04
NasNet-Mobile	83.45 ± 7.36	84.81 ± 2.19	81.90 ± 17.46	0.85 ± 0.05	0.94 ± 0.04
NasNet-Large	85.23 ± 8.25	79.32 ± 16.28	91.90 ± 5.77	0.84 ± 0.10	0.93 ± 0.05

**Table 3:** Classification results without data augmentation.

CNN model	Accuracy (%)	Sensitivity (%)	Specificity (%)	$F_1$ score	AUC
AlexNet	86.85 ± 13.66	80.25 ± 22.49	94.29 ± 4.84	0.85 ± 0.16	0.94 ± 0.04
GoogLeNet	93.83 ± 6.97	96.71 ± 4.06	90.57 ± 10.53	0.94 ± 0.06	0.96 ± 0.04
SqueezeNet	87.52 ± 6.45	86.84 ± 10.11	88.29 ± 12.01	0.88 ± 0.06	0.94 ± 0.06
ShuffleNet	95.97 ± 5.09	95.44 ± 7.47	96.57 ± 2.96	0.96 ± 0.05	0.97 ± 0.03
ResNet-18	95.44 ± 8.02	98.99 ± 1.65	91.43 ± 15.25	0.96 ± 0.07	0.98 ± 0.03
ResNet-50	93.62 ± 6.17	95.57 ± 6.27	91.43 ± 6.06	0.94 ± 0.06	0.98 ± 0.02
ResNet-101	93.29 ± 5.69	96.20 ± 1.79	90.00 ± 10.10	0.94 ± 0.05	0.98 ± 0.02
Xception	91.11 ± 10.14	89.56 ± 12.55	92.86 ± 7.80	0.91 ± 0.10	0.96 ± 0.03
Inception-v3	93.62 ± 5.22	96.20 ± 0.00	90.71 ± 11.11	0.94 ± 0.07	0.97 ± 0.04
Inception-ResNet-v2	88.59 ± 7.59	89.24 ± 2.69	87.86 ± 13.13	0.89 ± 0.07	0.96 ± 0.05
VGG-16	89.26 ± 8.80	92.83 ± 6.24	85.24 ± 14.45	0.90 ± 0.08	0.96 ± 0.03
VGG-19	90.16 ± 7.72	87.34 ± 10.36	93.33 ± 5.77	0.90 ± 0.08	0.97 ± 0.03
DenseNet-201	96.20 ± 4.95	95.78 ± 5.27	96.67 ± 4.59	0.96 ± 0.05	0.98 ± 0.03
MobileNet-v2	95.97 ± 7.18	96.71 ± 6.04	95.14 ± 8.55	0.96 ± 0.07	0.97 ± 0.05
NasNet-Mobile	89.26 ± 8.14	91.56 ± 5.12	86.67 ± 13.27	0.90 ± 0.07	0.95 ± 0.06
NasNet-Large	88.59 ± 7.59	90.51 ± 0.90	86.43 ± 17.17	0.90 ± 0.06	0.96 ± 0.03

For the case of training the networks without data augmentation, DenseNet-201, MobileNet-v2, ShuffleNet, and ResNet-18 provide the average accuracy > 95%, while DenseNet-201 achieves the highest average accuracy (96.20%). GoogLeNet, ShuffleNet, ResNet-18, ResNet-50, ResNet-101, Inception-v3, DenseNet-201, and MobileNet-v2 result in the average sensitivity > 95%, while ResNet-18 has the highest average sensitivity (98.99%). ShuffleNet, DenseNet-201, and MobileNet-v2 provide the average specificity > 95%, while DenseNet-201 gives the highest average specificity (96.67%). ShuffleNet, ResNet-18, DenseNet-201, and MobileNet-v2 result in the highest average $F_1$ score = 0.96. The top three CNNs that achieve sensitivity > 95%, specificity > 95%, and $F_1$ score > 0.95 are ShuffleNet, DenseNet-201, and MobileNet-v2. DenseNet-201 is the best model for the classification of COVID-19 CT data. The networks that have the highest average AUC (0.98) are the ResNet family and DenseNet-201.

The results obtained from CNNs without data augmentation are all better than those with data augmentation in terms of accuracy (compare results between Tables 2 and 3). For the case of training the networks with data augmentation, models that have average accuracy > 90% are ResNet-18 (90.16%), ResNet-50 (92.62%), Inception-v3 b(91.28%), and DenseNet-201 (91.72%); models that have average sensitivity > 90% are SqueezeNet (91.56%), ResNet-50 (91.14%), Xception (90.72%), Inception-v3 (90.30%), VGG-19 (90.72%), and MobileNet-v2 (95.78%); models that have average specificity > 90% are ResNet-18 (90.95%), ResNet-50 (94.29%), ResNet-101 (98.10%), Inception-v3 (92.38%), DenseNet-201 (95.24%), and NasNet-Large (91.90%); and models that have average $F_1$ score > 0.9 are ResNet-18 (0.91), ResNet-50 (0.93), Inception-v3 (0.92), and DenseNet-201 (0.92). Two CNNs using data augmentation that have accuracy > 90%, sensitivity > 90%, specificity > 90%, and $F_1$ score > 0.9 are ResNet-50 and Inception-v3. The networks without data augmenttaion have higher or equal values for the average AUC than or to those with data augmentation.

**Figure 2:**
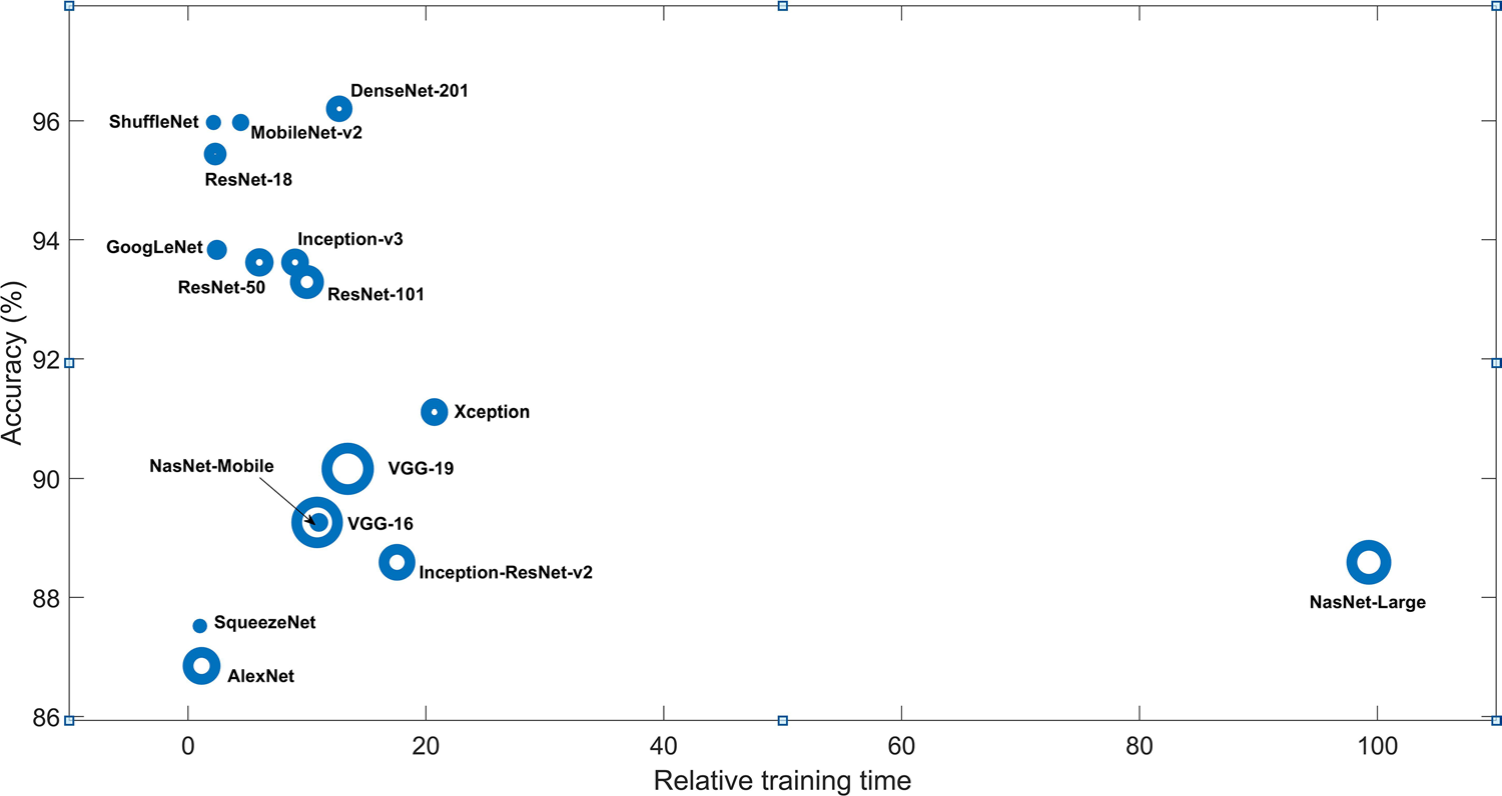
Plots of accuracy vs. relative training time (ratio of training time of a network to the training time of the SqueezeNet) of 16 pretrained CNNs using COVID-19 CT database, where the circle size indicates the magnitude of memory in MB.

In summary, without data augmentation, the best classifier is DenseNet-201, which has the best accuracy, best balance between sensitivity and specificity, top $F_1$ score, and top AUC. Figure 2 shows the plot of accuracy versus relative training time obtained from the 16 pretrained CNNs without data augmentation.

## Discussion

The benchmark results using the same database reported in^12^, with a fixed split data of about 80% for training and 20% for testing, have accuracy = 84.7%, sensitivity = 76.2%, and $F_1$ score = 0.85, using a fine-tuned pretrained DenseNet with data augmentation. The results obtained from the 16 CNNs without data augmentation are better than these benchmark results.

The study published in^13^ applied 10 pretrained CNN using a different COVID-19 database with the same ratio of training and testing data, which is not publicly available, reported among all the 10 networks, ResNet-101 was the best classification model. ResNet-101 achieved accuracy = 99.51%, sensitivity = 100%, and specificity = 99.02%. Although using a different database, the results obtained in this study are comparable. However, the input data processing and training reported in^13^ requires much effort by requiring the extraction of regions of interest by a radiologist, which is subjective, time-consuming, and likely hinders the real-time application of the pretrained networks.

The work reported in^14^ requires the pre-processing of 3D CT scans by extracting the regions of interest using a U-Net for image segmentation. The pre-processed images were then passed to the COVNet for the prediction. The sensitivity and specificity obtained from COVNet were 87% and 92%, respectively, using a dataset that is not publicly available. Another work on the classification of COVID-19 CT images collected from 259 patients reported in^15^ modified the pretrained Inception that achieved accuracy = 79.3%, sensitivity = 67%, and specificity = 83%, and another test achieving accuracy = 85.2%. Similarly, the input images are extracted regions of interest such as small patchy shadows and interstitial changes, multiple ground glass and infiltrates in both lungs. The study reported in^16^ used the concatenation of two pretrained ResNet-based networks and the Bayesian function for screening COVID-19 patients using CT imaging. The data pre-processing of classification procedure requires 3D segmentation, extraction of regions of interest (such as ground-glass appearance, striking peripheral distribution along with the pleura, and independent focus of infections), and data augmentation. The overall accuracy obtained was 86.7%. The classification results obtained in this study are preferable to those reported in^14–16^ in terms of accuracy and implementation of input data.

Although the use of regions of interest or cropped images is widely adopted for deep learning, including other classification problems^19–22^, this study finds that the direct input of CT images, which are then resized to fit the input size of the pretrained CNN, and transfer learning without data augmentation can achieve very high and better classification performance than those using data augmentation. Such findings are useful for the rapid deployment of AI tools to meet the urgent demand for curbing the pandemic, because it can relieve the task of manual detection of regions of interest carried out by experienced radiologists, employment of image segmentation methods, and more data collection.

**Figure 3:**
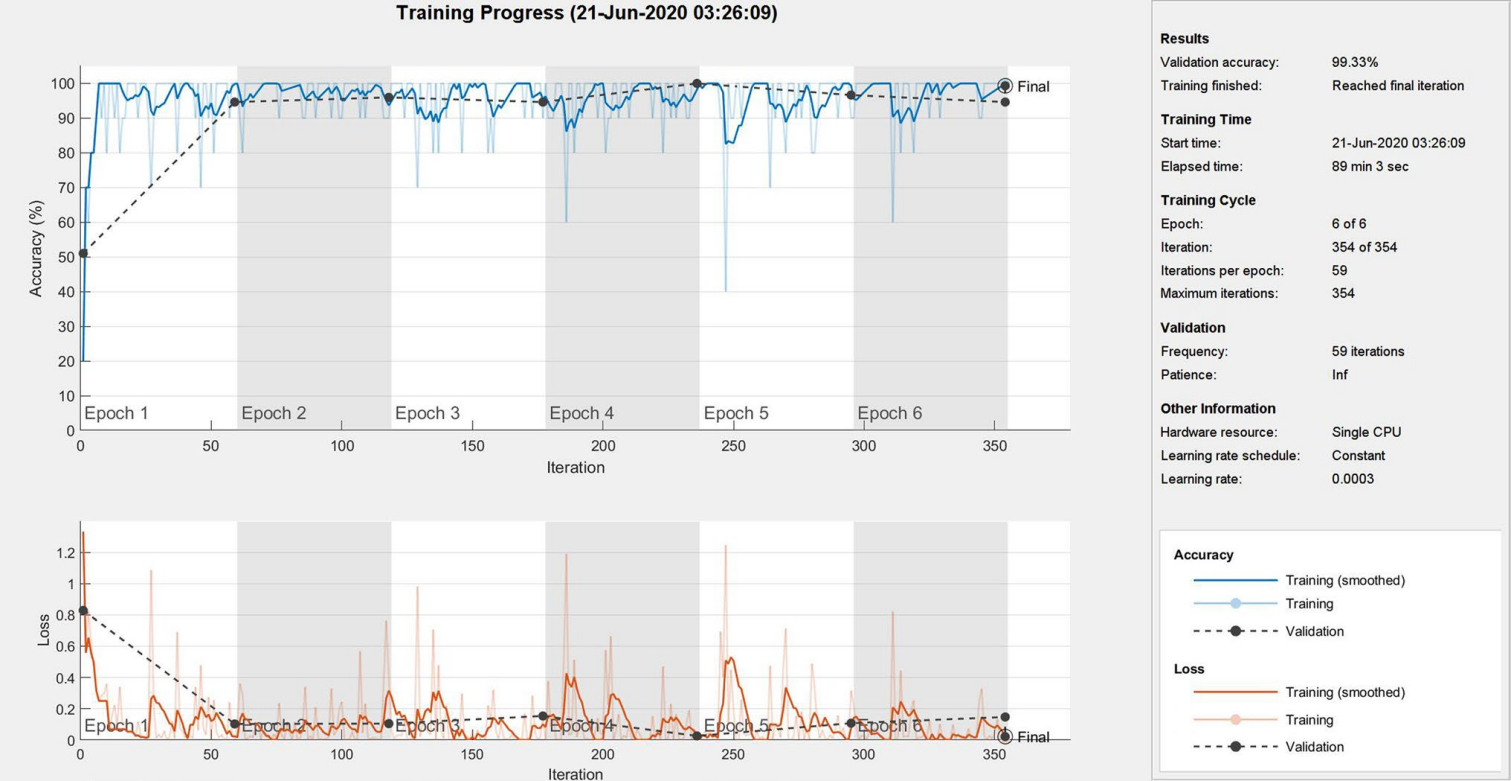
A training process of DenseNet-201.

**Figure 4:**
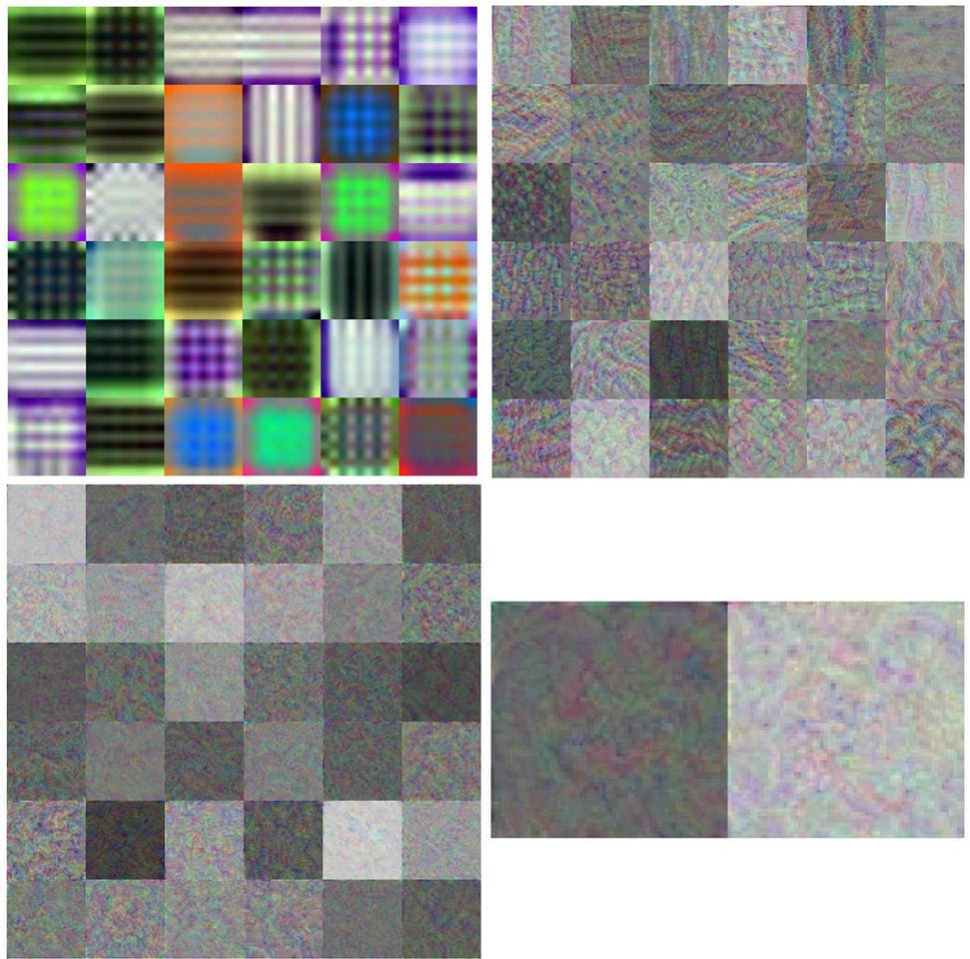
Features learned by DenseNet-201: 36 features in layer ’conv1|conv’(convolution) (top left), layer ‘conv4_block7_1_conv’ (convolution) (top right), layer ‘conv5_block9_1_conv’ (convolution) (bottom left), and 2 features in layer ‘new_fc’ (fully connected) (bottom right).

Using the described network-training configuration with only 6 epochs, the CNNs could provide a very high performance of classification. Figures 3 and 4 show one of the training processes of DenseNet-201 (best network) and some features obtained from the deep learning of the best network, respectively.

As the numbers of COVID-19 and non-COVID-19 CT images used in this study are 349 and 397, respectively, the binary classification in this study was not much disadvantaged from the class imbalance problem, where the class distributions are highly imbalanced. Due to imbalanced data, classifiers tend to result in low predictive accuracy for the minority class. Medical datasets are often not balanced in the class labels because of limited samples collected from patients and cost for acquiring annotated data. There are many techniques proposed for addressing class imbalance , which can be applied to medical imaging, such as the “deep domain adaptation”^23^ for handling the shortage of large amounts of labeled data, weighted loss method by updating the loss function to result in the same loss for all classes, downsampling by removing images from the majority class, and oversampling by adding more images to minority classes using artificial data augmentation^24,25^. Open challenges in imbalance data and exploration for solutions can be found in^26^.

## Conclusions

AI-based medical diagnosis systems based on deep learning of medical imaging are increasingly recognized to be clinically useful. However, development of suitable deep-learning networks and effective training strategy for clinical applications is a topic of research that needs to be explored^27^. Through a comprehensive investigation of 16 pretrained CNNs using certain parameter specification and training strategy for the networks, this study discovers the very high performance of several of these networks for COVID-19 diagnosis using CT images. The network configuration of the pretrained models can be implemented for classification of other image modality, such as X-ray, for the detection of COVID-19.

Most AI studies on chest CT used for differentiating COVID-19 pneumonia from other causes of pneumonia consider both three-class classification problems (COVID-19 pneumonia, non-COVID-19 pneumonia, and healthy) and two-class classification (COVID-19 pneumonia and healthy)^2^. Due to the limit of publicly available data, this study concerns with the two-class classification. However, extension of the use of pretrained CNNs to the three-class classification of COVID-19 imaging data is straightforward.

The findings reported from this study bring benefits to the development of fast and efficient diagnostic tools using imaging data and contribute to further leading into the development of more accurate point-of-care diagnostic and detection tools for containing the coronavirus pandemic.

## Data Availability

The MATLAB code used in this study is available at the author’s personal homepage: https://sites.google.com/view/tuan-d-pham/codes.
